# Non-contiguous finished genome sequence and description of *Salmonella enterica subsp. houtenae* str. RKS3027

**DOI:** 10.4056/sigs.3767427

**Published:** 2013-05-25

**Authors:** Songling Zhu, Hong-Liang Wang, Chunxiao Wang, Le Tang, Xiaoyu Wang, Kai-Jiang Yu, Shu-Lin Liu

**Affiliations:** 1 Genomics Research Center of Harbin Medical University, Harbin, China; 2 Genetic Detection Center of First Affiliated Hospital, Harbin Medical University, Harbin, China; 3 Department of Critical Care Medicine, The Second Affiliated Hospital of Harbin Medical University, Harbin, China; 4 Department of Microbiology and Infectious Diseases, University of Calgary, Calgary, Canada

**Keywords:** *Salmonella enterica*, subspecies, *houtenae*, genome

## Abstract

*Salmonella enterica subsp. houtenae* serovar 16:z4, z32:-- str. RKS3027 was isolated from a human in Illinois, USA. *S. enterica subsp. houtenae* is a facultative aerobic rod-shaped Gram-negative bacterium. Here we describe the features of this organism, together with the draft genome sequence and annotation. The 4,404,136 bp long genome (97 contigs) contains 4,335 protein-coding gene and 28 RNA genes.

## Introduction

*Salmonella* is an important genus of human and animal pathogens [1], and more than 2,600 different serovars have been described. Currently, the genus *Salmonella* is divided into two species, *S. enterica,* and *S. bongori* [2]. *S. enterica* comprises seven subspecies: I (also called subspecies *enterica*), II (also called subspecies *salamae*), IIIa (also called subspecies *arizonae*), IIIb (also called subspecies *diarizonae*), IV (also called subspecies *houtenae*), VI (also called subspecies *indica*), and VII [3]. Most of *Salmonella* serovars belong to the *S. enterica* subspecies I and are responsible for disease in warm-blooded animals and humans [4]. Other serovars were usually isolated from cold-blooded organisms and the environment, but could also cause human disease occasionally. In contrast with *S. enterica* subspecies I, very limited information is available regarding pathogenicity of the other subspecies. When infecting humans, these serovars usually cause an intestinal infection (e.g., diarrhea), but previous reports in the literature [5] have shown that the serovars of *Salmonella* subspecies II–IV are capable of causing serious infections, including septicemia and abscesses. There has been an increase in case reports on extraintestinal infections caused by these subspecies [6]. *S. enterica subsp. houtenae* serovar 16:z4,z32:-- str. RKS3027 is a human isolate. This strain is of interest because of its pathogenicity as well as its divergent phylogenetic position among *S. enterica*.

## Classification and features

**Figure 1:**
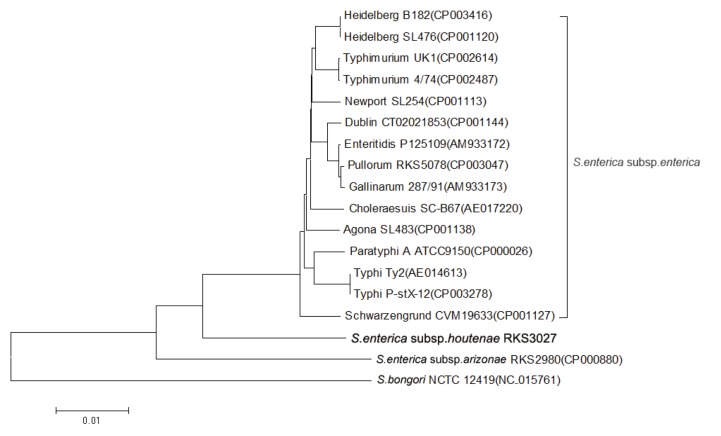
Phylogenetic tree highlighting the position of *S. enterica subsp. houtenae* strain RKS3027 relative to the other types and strains of *Salmonella*. GenBank accession numbers are indicated in the parentheses. The tree was built based on the comparison of concatenated nucleotide sequences of 2,500 orthologs conserved in all strains. Individual orthologous sequences were aligned by the MAFFT [9] and phylogenetic tree was constructed by using the neighbor-joining method within the MEGA software [10].

Few 16S rRNA sequences of *Salmonella* subspecies are available except *S. enterica* subsp. *enterica*. Meanwhile, it is increasingly commonplace to construct the phylogenetic tree by using the whole-genome sequence for higher precision and robustness [7,8]. Therefore we used a total of 2,500 orthologs of 18 strains of *Salmonella* for constructing a genome-scale phylogenetic tree. Genetic relatedness of *S. enterica subsp. houtenae* strain RKS3027 to other *Salmonella* subspecies strains was shown in Figure 1. On the tree, all *S. enterica* subsp. *enterica* strains were clustered together, and *S. enterica subsp. houtenae* RKS3027 positioned between *S. enterica* subsp. *enterica* and *S. bongori*.

The *Salmonella* genus belongs to the bacterial family *Enterobacteriaceae* [11]. The bacteria are rod shaped, Gram-negative, with diameter of 0.7 to 1.5 µm and length of 2 to 5 µm (Table 1). They are facultative anaerobes, non-spore-forming, flagellated, and motile. They grow within the optimal temperature range 35 °C - 37 °C and within an optimal pH range of 7.2-7.6. *S. enterica subsp. houtenae* is salicin-positive and able to grow in KCB medium, two distinguishing characteristics when compared with *S. enterica* subsp. *enterica*. The strain is deposited in the *Salmonella* Genetic Stock Centre (SGSC), University of Calgary, Canada as *S. enterica subsp. houtenae* RKS3027 (= SGSC 3086).

**Table 1:** Classification and general features of *S. enterica subsp. houtenae* RKS3027 according to the MIGS recommendations [12]

**MIGS ID**	**Property**	**Term**	**Evidence code**^a^
	Current classification	Domain *Bacteria*	TAS [13]
		Phylum *Proteobacteria*	TAS [14]
		Class *Gammaproteobacteria*	TAS [15,16]
		Order *Enterobacteriales*	TAS [17]
		Family *Enterobacteriaceae*	TAS [18-20]
		Genus *Salmonella*	TAS [18,21-23]
		Species *Salmonella enterica*	TAS [23,24]
		Subspecies *Salmonella enterica subsp. houtenae*	TAS [23,24]
		Strain RKS3027	IDA
		Serovar 16:z3, z32:--	IDA
	Gram stain	Negative	IDA
	Cell shape	Rod-shaped	IDA
	Motility	Motile	IDA
	Sporulation	Non-sporulating	IDA
	Temperature range	Mesophilic	IDA
	Optimum temperature	35 °C - 37 °C	IDA
	Carbon source	Glucose	IDA
	Energy source	Chemoorganotrophic	IDA
MIGS-6	Habitat	Reptiles	IDA
MIGS-6.3	Salinity	Medium	IDA
MIGS-22	Oxygen	Facultative anaerobes	IDA
MIGS-15	Biotic relationship	Endophyte	IDA
MIGS-14	Pathogenicity	Pathogenic	IDA
MIGS-4	Geographic location	Illinois, USA	NAS
MIGS-5	Sample collection time	1986	NAS
MIGS-4.1	Latitude	Not report	NAS
MIGS-4.2	Longitude	Not report	NAS
MIGS-4.3	Depth	Not report	NAS
MIGS-4.4	Altitude	Not report	NAS

a) Evidence codes - IDA: Inferred from Direct Assay; TAS: Traceable Author Statement (i.e., a direct report exists in the literature); NAS: Non-traceable Author Statement (i.e., not directly observed for the living, isolated sample, but based on a generally accepted property for the species, or anecdotal evidence). These evidence codes are from the Gene Ontology project [25].

## Genome sequencing information

### Genome project history

This organism was selected for sequencing on the basis of its phylogenetic position and its serious virulence in humans compared to the reptiles. This Whole Genome Shotgun project has been deposited at DDBJ/EMBL/GenBank under the accession ANHR00000000. The version described in this paper is the first version, ANHR01000000, and the sequence consists of 97 large contigs. Table 2 presents the project information and its association with MIGS version 2.0 compliance [12].

**Table 2:** Project information

**MIGS ID**	**Property**	**Term**
MIGS-31	Finishing quality	Draft
MIGS-28	Libraries used	Illumina Paired-End library
MIGS-29	Sequencing platforms	Illumina HiSeq 2000
MIGS-31.2	Fold coverage	100 ×
MIGS-30	Assemblers	SOAPdenovo v1.05
MIGS-32	Gene calling method	RAST
	Genbank ID	ANHR00000000
	GOLD ID	Gi21447
	Project relevance	Evolution in bacteria, human pathogen

### Growth conditions and DNA isolation

*S. enterica subsp. houtenae* strain RKS3027 was grown Luria Broth (LB) medium at 37°C. The DNA was extracted from the cell, concentrated and purified using the Qiamp kit (Qiagen), as detailed in the manual for the instrument.

### Genome sequencing and assembly

The genome of *S. enterica subsp. houtenae* RKS3027 was sequenced using the Illumina sequencing platform by the paired-end strategy (2×100bp). The details of library construction and sequencing can be found at the Illumina web site [26]. The final coverage reached 100-fold for an estimated genome size of 4.5 Mb. The sequence data from Illumina HiSeq 2000 were assembled with SOAPdenovo v1.05. The final assembly contained 97 large contigs (>3000 bp) in 59 scaffolds generating a genome size of 4.4 Mb.

### Genome annotation

Genes were predicted using RAST (Rapid Annotation using Subsystem Technology) [27] with gene caller GLIMMER3 [28] followed by manual curation. The predicted bacterial protein sequences were compared with the annotated genes from four available *Salmonella* genomes, i.e., *S. enterica* subsp*. enterica* Typhi P-stx-12, *S. enterica* subsp*. enterica* Heidelber*g* B182, *S. enterica* subsp*. enterica* Typhimurium UK-1 and *S. enterica* subsp*. enterica* Typhimurium 4/74 and searched against the Clusters of Orthologous Groups (COG) databases using BLASTP. The BLAST results were filtered with the following parameters: identities >90% and compared length >70%. CGViewer was used for visualization of genomic features [29].

## Genome properties

**Figure 2:**
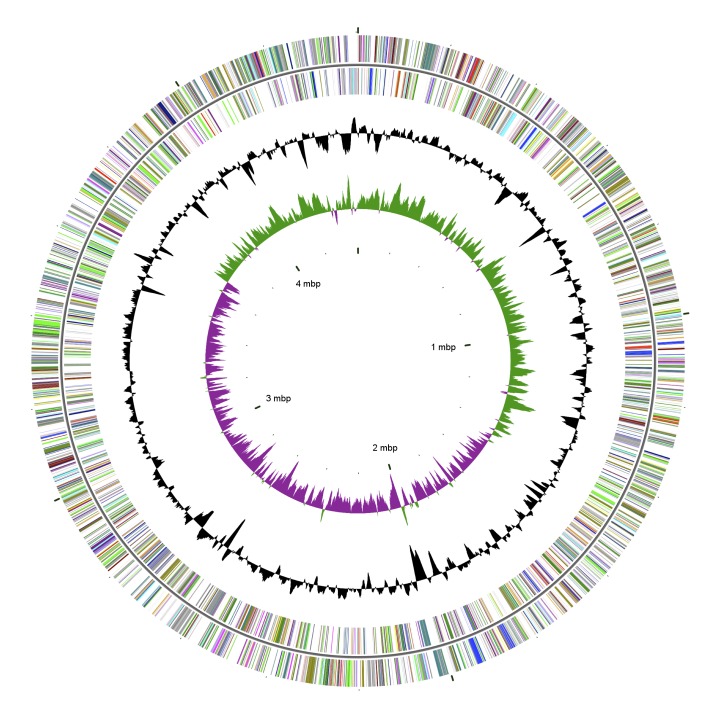
Graphical circular map of the *S. enterica subsp. houtenae* strain RKS 3027 genome. From the outside to the center: genes on forward strand (color by COG categories), genes on reverse strand (color by COG categories), GC content, GC skew. The map was generated with the CGviewer software.

The genome of *S. enterica subsp. houtenae* RKS3027 is 4,404,136 bp long (97 contigs) with a 51.68% G + C content (Table 3 and Figure 2). Of the 4,363 predicted genes, 4,335 were protein-coding genes, and 28 were RNAs (1 5S rRNA gene and 27 predicted tRNA genes). A total of 3,378 genes (77.42%) were assigned a putative function. The remaining genes were annotated as hypothetical proteins. The properties and statistics of the genome are summarized in Table 3. The distribution of genes into COGs functional categories is presented in Table 4.

**Table 3:** Nucleotide content and gene count levels of the genome

**Attribute**	Value	% of total^a^
Genome size (bp)	4,404,136	
DNA coding region (bp)	3,824,952	86.85
DNA G+C content (bp)	2,276,005	51.68
Total genes	4,363	100
RNA genes	28	0.06
Protein-coding genes	4,335	99.36
Genes assigned to COGs	3,378	77.42

a) The total is based on either the size of the genome in base pairs or the total number of protein coding genes in the annotated genome.

**Table 4:** Number of genes associated with the 25 general COG functional categories

**Code**	**Value**	**%age**^a^	**Description**
J	163	3.76	Translation
A	1	0.02	RNA processing and modification
K	281	6.48	Transcription
L	176	4.06	Replication, recombination and repair
B	0	0.00	Chromatin structure and dynamics
D	32	0.74	Cell cycle control, mitosis and meiosis
Y	0	0.00	Nuclear structure
V	48	1.11	Defense mechanisms
T	103	2.38	Signal transduction mechanisms
M	235	5.42	Cell wall/membrane biogenesis
N	95	2.19	Cell motility
Z	0	0.00	Cytoskeleton
W	0	0.00	Extracellular structures
U	41	0.95	Intracellular trafficking and secretion
O	138	3.18	Posttranslational modification, protein turnover, chaperones
C	254	5.86	Energy production and conversion
G	343	7.91	Carbohydrate transport and metabolism
E	319	7.36	Amino acid transport and metabolism
F	77	1.78	Nucleotide transport and metabolism
H	131	3.02	Coenzyme transport and metabolism
I	89	2.05	Lipid transport and metabolism
P	175	4.04	Inorganic ion transport and metabolism
Q	47	1.08	Secondary metabolites biosynthesis, transport and catabolism
R	318	7.34	General function prediction only
S	312	7.20	Function unknown
-	957	22.08	Not in COGs

a) The total is based on the total number of protein coding genes in the annotated genome.
